# Transactions of Medical and Surgical Society of Baltimore

**Published:** 1877-09

**Authors:** 


### Reports of Societies.

---

### TRANSACTIONS OF MEDICAL AND SURGICAL SOCIETY OF BALTIMORE.

---

[EXTRACT.]

"Relations of cases" being in order, the following were given to the Society:

CASE I. Dr. Jno. N. Monmonier, had been called to see a child eight years of age, who was bleeding profusely from a wound of the tongue. The list of styptics having been exhausted before his arrival, without benefit, he decided to resort to operative interference, viz: to pass sutures upon either side of the wound, draw it together and stuff the interstices firmly with lint, and tie the sutures over the compress thus formed. By this means direct pressure upon the bleeding vessels was obtained, and the hemorrhage controlled. The sutures were removed the next morning and the case did nicely.

Case II. Dr. Thos. R. Brown brought before the Society a patient, upon whom he had operated, with the following history:—X. Y., aged fifty-five, received about one year ago, a blow over the right inferior maxilla, with a piece of iron; soon after a swelling of the gum was noticed, and two teeth were extracted by a dentist, who supposed the swelling to be the result of a dental abscess. The tumor continued to grow, however, and Prof. Smith who saw it diagnosed it an epulis. The case came subsequently under the notice and care of Prof. Brown, who, regarding it as malignant, advised its removal, which was effected by an incision commencing to the left of the median line of the chin, descending by a curved line to the inferior margin of the maxilla, and passing backward to the ramus. The bone was then nicked with a saw and the diseased portion was broken away. The flap was restored to its place, and, notwithstanding the extent of the surface made bare, the wound healed by first intention. The line of incision is hidden by the beard, and the patient suffers little or no deformity.

Case III. Dr. A. B. Arnold stated that he had had recently, in one family, five cases of diphtheria, occurring consecutively; one of these he lost, and in this case the disease had *invaded the larynx.* The other cases, although very severe, were free from this complication. The point he desired to make was, that when the larynx is free from the diphtheritic invasion the disease is not so dangerous.

Dr. Lynch stated that his experience confirmed this view, and that the establishment of cinchonism early in the history of the case is *very frequently preventive* of the laryngeal complication. Since his adoption of the quinine treatment his proportion of fatal cases has been much smaller.

Case IV. Dr. C. C. McDowell relates a case of scleroderma occurring in a patient three weeks of age. The child had been from birth subject to occasional attacks of partial pulmonary collapse, and after the establishment of the dermal lesion, it was always exascerbated by an attack of the pulmonary collapse, so that the two diseasses seemed to bear a relation to one another—perhaps that of cause and effect. The cutaneous surface was largely involved. During the pulmonary attacks warm baths, artificial respiration and stimulants, were resorted to; in the intervals an inunction of iodine and iodide of potassium was used. Four or five weeks of treatment were necessary and the child gradually recovered.

Case. V. Dr. Arnold related a case of chorea which had resisted all usual forms of treatment for two and a half years, but which yielded to enforced *absolute rest* of the voluntary muscles, *in bed*, combined with alternated hot and cold douches to the spine. (Iron was also used against the concomitant animia.)

